# Treatment approaches, outcomes and prognostic indicators in patients with tinnitus and temporomandibular disorders evaluated with DC/TMD: A systematic review and Meta‐analysis

**DOI:** 10.1111/joor.13796

**Published:** 2024-07-17

**Authors:** d’ Apuzzo Fabrizia, Minervini Giuseppe, Cerbone Martina, Rotolo Rossana Patricia, Grassia Vincenzo, Nucci Ludovica

**Affiliations:** ^1^ Multidisciplinary Department of Medical‐Surgical and Dental Specialties University of Campania Luigi Vanvitelli Naples Italy

**Keywords:** cervico‐mandibular manual therapy, conservative orofacial therapy, DC/TMD, orofacial pain, temporomandibular disorders, tinnitus

## Abstract

**Objective:**

This systematic review summarised the results reported in randomised controlled trials (RCTs) aimed at evaluating the different treatment approaches in patients with tinnitus associated with temporomandibular disorders (TMD) evaluated with the diagnostic criteria of temporomandibular disorders (DC/TMD), and the possible predictive factors influencing treatment outcomes.

**Methods:**

The electronic databases Medline, Web of Science, Latin American and Caribbean Health Sciences Literature (LILACs) were searched systematically up to March 2023. Only RCTs with full texts were included in this study. The eligibility of the RCTs selected was based on the PICO model (participants, intervention, comparators, outcomes), and subjects of any age, sex or ethnicity, were included when showing both tinnitus and TMD, diagnosed through DC/TMD criteria.

**Results:**

From a total of 635 articles, only five RCTs were included and the data from a total of 329 participants were examined. Two RCTs focused on the efficacy of the multidisciplinary approach among people with tinnitus who have TMD; two RCTs examined prognostic indicators predicting a positive outcome after multidisciplinary orofacial treatment; one RCT analysed low‐level laser therapy's effectiveness with Nd:YAG laser.

**Conclusion:**

A multidisciplinary non‐invasive approach is the most efficacious treatment for tinnitus in patients diagnosed with TMD. Baseline tinnitus severity, gender, quality of life, age, and tinnitus duration were considered predictive factors of clinical outcomes in patients diagnosed with DMTs and referring tinnitus.

## Graphical abstract

In the five selected studies the best treatment approach is multidisciplinary and non‐invasive. The predictive factors of TMD in patients with Tinnitus is its severity and duration, gender, quality of life and age.

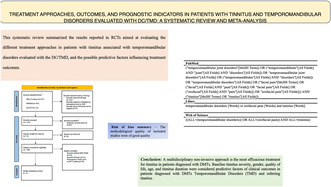

## 1 INTRODUCTION

The subjective conscious impression of a sound or noise, intermittent or pulsating, without any external auditory source, is called tinnitus.^1^ Tinnitus can be described as unilateral or bilateral, or emerging within the head.^2^

The prevalence ranges between 5% and 15% of the adult population. In children, tinnitus seems not to be perceived while the prevalence rate can reach 30% among the elderly developing into a chronic disease that can cause stress, mental illness, insomnia and poor quality of life.^3^ It is a significant discomfort for the patients, and a mysterious occurrence for audiologists and mental health specialists.^4^ The aetiology of tinnitus is multifactorial and, thus, challenging to be defined.^5^ Possible causes include hyperactivity of the auditory system induced by peripheral denervation and lack of auditory input, even if this association is not always direct.^5^, ^6^ Among the several risk factors^7^ that can be mentioned there are obesity, smoking, alcohol, injuries involving the head, arthritis and hypertension.^1^, ^8^ Tinnitus can be stimulated by a few drugs, that is, salicylates, aminoglycoside antibiotics, and some of antineoplastic agents, particularly platinum‐based ones.^1^ Two‐thirds of tinnitus sufferers can change their volume and pitch during somatic movements such as holding their neck muscles taut or clenching their jaw.^9^ The tinnitus in question is referred to as ‘somatosensory tinnitus’.^6^ Since tinnitus is generally subjective, patients' self‐reports are the primary source of information for its diagnosis.^10^ The Tinnitus Handicap Inventory (THI), a self‐report questionnaire^11^ is one of the most crucial tools to assess the complexity of tinnitus patients' issues.^4^

Many correlations have been found between different aural symptoms (i.e. vertigo, ear pain, impaired hearing) with temporomandibular disorders (TMD).^12^, ^13^

There are also numerous comorbidities related to tinnitus, particularly anxiety, depression and dysfunction of the temporomandibular joint (TMJ).^1^, ^14^

About this scenario, several hypotheses have been put forward to explain the presence of otologic signs in TMD patients, for example, from an embryological point of view, the middle ear and the masticatory muscles are similar; compressions of the vessels, nerves and ligaments of the ear region induced by movements of the mandibular condyle have also been studied.^12^ Moreover, this relationship could be also explained by the presence in both anatomical structures of cranial nerves such as the trigeminal (V) and facial (VII).^12^

The term ‘temporomandibular disorders’ is used to indicate a group of diseases that involve TMJ and/or masticatory muscles, and associated structures.^15^, ^16^, ^17^ During the clinical examination the most reported symptoms by patients are pain in the jaw and/or face, clicking and crepitus, limited or painful mouth opening, difficulties during chewing, and stiffness in the jaw muscles.^18^ One of the most prevalent painful non‐dental orofacial diseases affecting adults, adolescents and children is TMD.^6^ About 60%–70% of the general population complains of at least one symptom of TMD,^19^ and this may increase the possibility of developing tinnitus.^12^

Over the years, many multidisciplinary conservative treatments showed^20^, ^21^ a good impact on tinnitus control with individualised approaches for each patient's specific tinnitus subtype and characteristics.^6^, ^22^ Multimodal treatments, including behavioural and educational methods, appeared to provide greater effectiveness than a single treatment.^23^, ^24^ However, the use of non‐homogeneous diagnostic criteria, lacking information on patients' medical conditions, and lacking research methodologies with limited weak validity of available data, highlighted the need for additional research with an effective methodological approach.^25^

Thus, the primary objective of this systematic review was to evaluate patients diagnosed with tinnitus associated with TMD, assessed through the Research/Diagnostic Criteria for TMD,^15^ and the different treatment approaches with related outcomes. The secondary objective was to check the possible predictive factors influencing treatment results in the different groups of patients.

## 2 MATERIALS AND METHODS

### 2.1 Protocol and registration

The current systematic review protocol was created a priori according to the Cochrane Handbook for Systematic Reviews of Interventions 5.1.0^26^ and followed the guidelines reported in the Preferred Reporting Items for Systematic Reviews and Meta‐Analyses (PRISMA).^27^ The systematic review was recorded on the International prospective register of systematic reviews (PROSPERO) and approved with the number CRD42022314972 in April 2022.

### 2.2 Eligibility criteria

Based on the following participant, intervention, comparison, and outcome (PICO) model, the eligibility of the randomised controlled trials (RCTs) selected for this study was evaluated:

(P) Subjects of any age, sex, or ethnicity, were included when showing both tinnitus and temporomandibular disorders, diagnosed through DC/TMD or Research DC/TMD criteria^15^;
(I) Intervention consisted of different treatment protocols used to treat tinnitus and TMD (e.g. orofacial physical therapy, craniocervical and TMJ exercise, self‐massage on the masticatory muscles, patient education, relaxation therapy, and low‐level laser therapy);
(C) Comparison included physical therapy/education alone, a delayed treatment;
(O) Outcome measures consisted in the evaluation of the different treatment approaches, and in the assessment of any prognostic indicators of the treatment outcomes. No limitations concerning language, publication year, or status were applied. Only RCTs with full texts were included in this study.

Excluded studies included those involving animals, patients with medical causes of tinnitus (i.e. infections, otosclerosis, Ménière's disease), other correlated syndromes, otological or craniofacial cancers, previous maxillofacial surgery, subjects with incomplete medical records. Cross‐sectional surveys, case reports or series, reviews and meta‐analyses, text and opinion papers, editorials, replies to the author/editor, interviews, book chapters, and protocols were not considered.

### 2.3 Information sources

The electronic databases Medline, Web of Science, Latin American and Caribbean Health Sciences Literature (LILACs) were searched systematically without restrictions for publication date, language or type up to 14 March, 2023, following the approach presented by Table 1. Additionally, a manual search of earlier systematic reviews on the same subject was done in their references' list.

**TABLE 1:** Search strategy.

PubMed
("temporomandibular joint disorders"[MeSH Terms] OR ("temporomandibular"[All Fields] AND "joint"[All Fields] AND "disorders"[All Fields]) OR "temporomandibular joint disorders"[All Fields] OR ("temporomandibular"[All Fields] AND "disorders"[All Fields]) OR "temporomandibular disorders"[All Fields] OR ("facial pain"[MeSH Terms] OR ("facial"[All Fields] AND "pain"[All Fields]) OR "facial pain"[All Fields] OR ("orofacial"[All Fields] AND "pain"[All Fields]) OR "orofacial pain"[All Fields])) AND ("tinnitus"[MeSH Terms] OR "tinnitus"[All Fields])
Lilacs
temporomandibular disorders [Words] or orofacial pain [Words] and tinnitus [Words]
Web of Science
((ALL=(temporomandibular disorders)) OR ALL=(orofacial pain)) AND ALL=(tinnitus)

### 2.4 Search strategy and study selection

The search strategy was performed independently by two reviewers and included the following main Medical Subject Headings (MeSH) terms and keywords: ‘Tinnitus’ AND ‘Temporomandibular disorders’ OR ‘Orofacial pain’. The two reviewers then separately evaluated the full‐text versions, and any differences of opinion regarding the eligibility of the included studies were settled by consensus. Studies that did not match the inclusion criteria in this selection phase were excluded.

### 2.5 Data extraction

Using a data collection on Microsoft Excel sheet, two independent researchers collected data from the included studies. In case of dispute, the opinion of another reviewer was considered to solve it. The following data were extracted: (1) Authors; (2) Publication year; (3) Country; (4) Sample; (5) Gender; (6) Mean age; (7) TMD diagnosis through DC/TMD; (8) diagnosis of Tinnitus; (9) Interventions; (10) Control; (11) Outcomes and predictive factors. Extracted data from the included studies are presented in Table 2. A meta‐analysis was performed on measurable data.

**TABLE 2:** Main characteristics of the studies included in the present systematic review.

Authors	Publication year	Country	Sample	Gender	Mean age	DC/TMD diagnosis	Tinnitus diagnosis	Intervention	Control	Outcomes and predictive factors
Delgado de la Serna et al.^32^	2020	Spain	Group exercise + education + manual therapy (*N* = 31) Group exercise + education (*N* = 30) Total: 61 patients	Group exercise + education + manual therapy M/F = 12/19 Group exercise + education M/F = 13/17 Total: 61 patients	42.5 44.0	Diagnosis of TMD according to the Research Diagnostic Criteria for TMD	Diagnosis of tinnitus attributed to TMD; that is, they had to report self‐reported tinnitus symptoms and have a diagnosis of TMD according to the Research Diagnostic Criteria for TMD	The intervention included a cranio‐cervical and TMJ exercise program, self‐massage of the masticatory muscles (masseter and temporalis), and patient education	Group exercise + education: same intervention without manual therapy	All outcomes were assessed at baseline (T0), 1 week after the treatment program (T1), and three (T2) and six (T3) months after. Primary outcomes: Intensity of TMD Pain (NPRS) (F = 10.639, *p* < .001, *η* ^2^_p_ = 0.153) Tinnitus Severity (VAS) (*F* = 17.878, *p* < .001, *η* ^2^_p_ = 0.233) Secondary outcomes: THI (*F* = 39.291, *p* < .001, *η* ^2^_p_ = 0.501) CF‐PDI (*F* = 18.096, *p* < .001, *η* ^2^_p_ = 0.395) SF‐12 (*F* = 0.590, *p* = .622, *η* ^2^_p_ = 0.01) BDI‐II (*F* = 14.234, *p* < .001, *η* ^2^_p_ = 0.194) Mandibular range of motion mouth opening: *F* = 17.683, *p* < .001, *η* ^2^_p_ = 0.367; lateral excursions: *F* = 18.594, *p* < .001, *η* ^2^_p_ = 0.395 PPTs in the masseter (*F* = 29.494, *p* < .001, *η* ^2^_p_ = 0.415), temporalis (*F* = 18.594, *p* < .001, *η* ^2^_p_ = 0.395), and the TMJ (*F* = 15.448, *p* < .001, *η* ^2^_p_ = 0.363)
Plaza‐Manzano et al.^33^	2021	Spain	Group exercise + education + manual therapy (*N* = 28) Group exercise + education (*N* = 28) Total: 56 patients	Group exercise + education + manual therapy M/F = 12/19 Group exercise + education M/F = 13/17 Total: 56 patients	42.5 44.0	All patients exhibited a diagnosis of TMD according to the Diagnostic Criteria for TMD (DC/TMD)	Diagnosis of tinnitus attributed to TMD, that is an association between both disorders had to be self‐reported by the own patient	The intervention included a cranio‐cervical and TMJ exercise program, self‐massage of the masticatory muscles (masseter and temporalis), and patient education	Group exercise + education: same intervention without manual therapy	All outcomes were assessed at baseline (T0), one week after the treatment program (T1), and three (T2) and six (T3) months after. The higher baseline scores of tinnitus severity predicted better outcomes at 3‐ and 6‐months post‐intervention (from 12% to 42% of the variance) for changes in tinnitus severity in both groups and for changes in tinnitus‐related handicap in the exercise/education group. The baseline pressure pain hypersensitivity over the temporalis muscle was consistently associated with poorer clinical outcomes at 3 and 6 months (explaining from 10% to 41% of the variance) in both groups
Van der Wal, Michiels et al.^28^	2020	Belgium	Early‐Start Group (*N* = 40) Delayed‐Start Group (*N* = 40) Total 80 patients	Early‐Start Group M/F = 18/22 Delayed‐Start Group M/F = 24/16 Total M/F = 42/38	Early‐Start Group 46 Delayed‐Start Group 45 Total 45	TMD diagnosed according to the Diagnostic Criteria for TMD (DC‐TMD)	Patients included with a combination of moderate to severe chronic subjective tinnitus, defined as a functional tinnitus index (TFI) score between 25 and 90 that had been stable for at least 3 months	The intervention consisted of orofacial physical therapy, comprising counselling regarding mouth habit reversal, bruxism, sleep hygiene, lifestyle advice and biofeedback; massage of the masticatory muscles; stretching exercises; and relaxation therapy	The delayed treatment design allowed us to obtain data for a control group by creating a waiting list, since the use of a control group that receives no treatment at all was not considered ethical in a tertiary center population	All outcome measures were documented at baseline, after 9 weeks in the delayed start group, immediately after the last treatment session (post‐treatment) and after 9 weeks of follow‐up. For the entire group, 34% of the patients showed a clinically relevant improvement of the TQ score (cutoff score ≥8.72 points) immediately after treatment. About the TFI score, 41% of the patients had a clinically relevant improvement (cutoff score ≥ 13 points). After the follow‐up period 46% (TQ) and 61% (TFI) reached the level of clinically relevant improvement compared to baseline, respectively
Van der Wal, Van de Heyning et al.^29^	2020	Belgium	Early‐Start Group (*N* = 61) Delayed‐Start Group (*N* = 40) Total 101 patients	Total %(M/F) = (42/38)%	Total 47	TMD diagnosed according to the Diagnostic Criteria for TMD (DC‐TMD)	Patients included with a combination of moderate to severe chronic subjective tinnitus, defined as a functional tinnitus index (TFI) score between 25 and 90 that had been stable for at least 3 months	The intervention consisted of orofacial physical therapy, comprising counselling regarding mouth habit reversal, bruxism, sleep hygiene, lifestyle advice and biofeedback; massage of the masticatory muscles; stretching exercises; and relaxation therapy	The delayed treatment design allowed us to obtain data for a control group by creating a waiting list, since the use of a control group that receives no treatment at all was not considered ethical in a tertiary center population	The multivariate logistic regression analysis, based on the clinically relevant change in TQ score after treatment, created a model comprising two characteristics: ‘duration of tinnitus’ and ‘a higher initial score on the TQ somatic subscale.’ This model correctly predicts the outcome on TQ in 68.5%. The multivariate binary logistic regression analysis, based on the clinically relevant change in TFI score after follow‐up, created a model consisting of three items: ‘age,’ ‘female gender,’ and ‘duration of tinnitus.’ This model correctly predicts the outcome on TFI in 68.1%
Demirkol et al.^30^	2017	Turkey	Nd:YAG laser (*N* = 15) 810 nm diode laser (*N* = 16) Total 31 patients	Nd:YAG laser M/F = 7/8 810 nm diode laser M/F = 10/6 Total M/F = 17/14	Nd:YAG laser 36.6 810 nm diode laser 40.1	TMD diagnosed according to the Research Diagnostic Criteria for Temporomandibular Disorders (RDC/TMD)	The severity of the tinnitus, the type of tinnitus and the frequency of the tinnitus were noted. Patients diagnosed with subjective tinnitus underwent a detailed assessment of TMDs	Nd:YAG laser and an 810 nm diode laser were used with a single‐probe laser handpiece parallel to the external auditory canal The energy density was set at 8 J/cm^2^, with 0.25 W output power. The probe diameters of Nd:YAG and 810 nm diode laser were 0.9 and 0.6 cm, and the focal spot areas were 0.625 and 0.282 cm^2^, respectively. LLLT was applied precisely and continuously into the external auditory meatus for 20 s for Nd:YAG laser and 9 sec for 810 nm diode laser. The laser parameters for the Nd:YAG laser irradiation were as follows: 0.25 W output power, 1000 l s pulse duration (VLP mode), 10 Hz frequency, 25 mJ pulse energy, 25 W peak power, and 8 J/cm^2^ energy density	The effectiveness of low‐level laser therapy with the Nd:YAG laser was compared with the use of the 810 nm diode laser	Statistically significant differences between the values at baseline and 1 month after treatment in the Nd:YAG laser (*p* = .001) and diode laser (*p* = .005) groups. The percentage improvement in tinnitus severity scores, according to the VAS and based on the median values, was 100% (group Nd:YAG) and 30% (group diode)

### 2.6 Risk of bias assessment

For this study, the risk of bias was evaluated by two researchers using version 2 of the Cochrane risk‐of‐bias tool for randomised trials. (RoB 2).^31^ Any disagreement was discussed until a consensus was reached with a third reviewer.

### 2.7 Statistical analysis

All the statistical analyses were performed with R 3.65 software (R foundation, Vienna, Austria). Mean difference (MD) and standard deviations (SD) were calculated on the sample data. A meta‐analysis was performed on the measurable data after different treatment modalities for tinnitus described in the RCTs versus controls. The odds ratio, risk ratio and risk difference were calculated, and the forest plots were used to display the results of a meta‐analysis assessing different treatment modalities.

## 3 RESULTS

### 3.1 Study selection and characteristics

**FIGURE 1:**
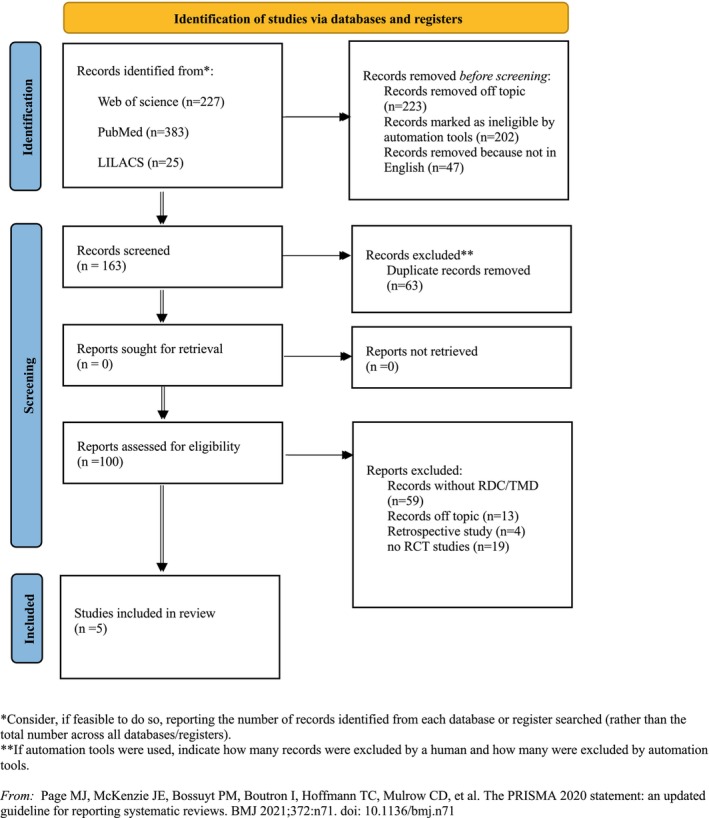
Prisma Flowchart. Consider, if feasible to do so, reporting the number of records identified from each database or register searched (rather than the total number across all databases/registers). **If automation tools were used, indicate how many records were excluded by a human and how many were excluded by automation tools. Adapted from Page et al.^34^

The initial search identified a total of 635 articles. Four hundred and seventy‐two studies were excluded before screening and 63 were duplicates. After title and abstract screening, 100 full texts were assessed by the reviewers considering the previous PICO model. Therefore, only five RCTs^28^, ^29^, ^30^, ^32^, ^33^ were included, according to the PRISMA 2020 flow diagram in Figure 1. The selected studies were released in the last 5 years (from 2017 to 2020). Of these, four RCTs were performed in Europe [two from Spain^32^, ^33^ and two from Belgium^28^, ^29^], and one from Turkey.^30^ Concerning the study designs, one was a multicenter, parallel group, randomised clinical study^32^; a secondary predictive analysis was used in one RCT^33^; a prospective and controlled represented as one RCT^30^; two RCTs were created using a delayed treatment design in a randomised controlled trial.^28^, ^29^

The data from a total of 329 participants were examined. The mean age of the selected participants in the five articles was 43.3 years. One hundred and seventy‐five patients were assigned to the intervention group, whereas 154 subjects were considered as a control group. Two RCTs^28^, ^32^ focused on the efficacy of the multidisciplinary approach among people with tinnitus who have TMD; two RCTs^29^, ^33^ examined prognostic indicators predicting a positive outcome after multidisciplinary orofacial treatment; only Demirkol et al.^30^ analysed low‐level laser therapy's effectiveness with laser Nd:YAG.

All outcome measures were recorded in different ways: in one RCT^32^ an assessor who was blind to group allocation evaluated all outcomes at baseline, 1 week after the therapy, 3 months following the last appointment for therapy, and 6 months after that; in one RCT^33^ the variation between scores at 3 and 6 months following intervention and scores at baseline was used to determine the changes on each clinical outcome; in two RCTs,^28^, ^29^ with a delayed treatment design, at baseline, after 9 weeks in the delayed start group, immediately following the last appointment for therapy, and after 9 weeks of follow‐up, all outcomes were recorded; in one RCT^30^ the score was calculated at baseline and 1 month following the final laser treatment.

As regards the method of randomization, in two RCTs^32^, ^33^ physical treatment alone or physical therapy combined with manual therapy was given to patients at random. The hidden attribution was conducted by a blinded external investigator using a computerised random array.^32^ In two others RCTs^28^, ^29^ patients were randomised into the early start group or delayed start group based on block‐randomization 1:1. The hidden attribution was generated with a Microsoft Excel spreadsheet by an expert statistician. Conversely, in one RCT,^30^ the randomization method was not explained. Table 2 recapitulates the key features of the RCTs selected for this systematic review. About blinding, all therapists involved worked blindly with patients.

### 3.2 Quality assessment and risk of bias

**FIGURE 2:**
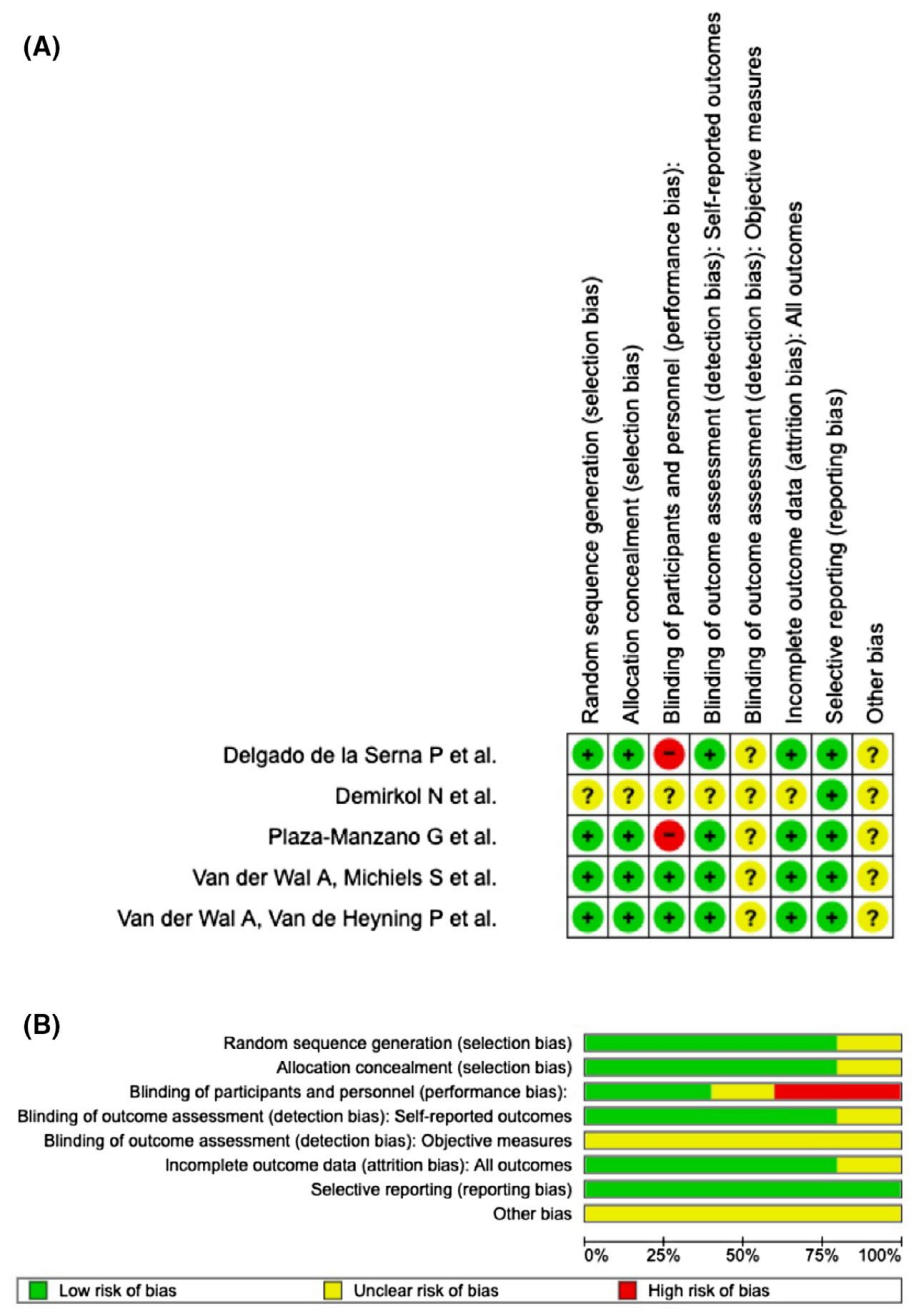
(A, B) Risk of bias domains.

RoB 2 of the selected RCTs are shown in Figure 2. Specifically, only Demirkol et al. showed ‘some concerns’ on all possible risks of bias, and a high risk of performance bias due to the blinding levels were found in two RCTs (Figure 2A). In terms of the randomization procedure, 80% of the research made sure there was low risk of bias. Only 40% of RCTs had a minimal risk of performance bias while 100% of them recorded every piece of outcome information (Figure 2B).

### 3.3 Treatment approaches

Delgado de la Serna et al.^32^ evaluated the impact on clinical results of adding some cervico‐mandibular manual therapies into an exercise and educational program in patients with TMD‐associated tinnitus. The sample consisted of 61 patients, whose 31 performed manual therapy group, exercise, and education, whereas the remaining 30 patients did not perform any therapy. It was analysed how interventions affected the intensity of TMD pain (numeric pain rating scale, NPRS), tinnitus severity (visual analogue scale, VAS), mandibular range of motion, pressure pain sensitivity, and the following different questionnaires: tinnitus handicap inventory (THI), craniofacial pain and disability inventory (CF‐PDI), short‐form survey (SF‐12), Beck depression inventory (BDI‐II). Patients receiving exercise/education with manual therapy showed greater improvements in TMD pain, tinnitus severity, overall TMD‐related disability (THI, and CF‐PDI), mandibular range of motion and PPT of the masticatory muscles, and depressive symptoms, compared to patients receiving only exercise/education. In contrast, both groups experienced similar changes in health‐related quality of life (SF‐12), and gender did not influence the outcomes. More details are shown in Table 2.

Van der Wal, Michiels et al. (2020)^28^ estimated the effectiveness of conservative orofacial therapy in 80 patients with somatosensory temporomandibular tinnitus (TST) by calculating the differences in changes on the Tinnitus Questionnaire (TQ), and Tinnitus Functional Index (TFI) from baseline to Week 9 of the study between the early start and delayed start groups (divided in 40 patients randomly selected in each group). The TQ and TFI change scores were selected using a cutoff score ≥8.72 points for the TQ and a cutoff score ≥ 13 points for the TFI. After receiving orofacial therapy, 34% and 46% of patients immediately after treatment and after follow‐up showed a clinically relevant change in the TQ score, respectively. Regarding the TFI score, 41% of the patients reported a clinically relevant increase immediately after treatment and 61% after follow‐up. This variation could be a consequence of the different characteristics between the two questionnaires.

Demirkol et al. (2017)^30^ planned this RCT to examine the therapeutic efficacy of low‐level laser therapy (LLLT) on the external acoustic meatus of TMD patients presenting with subjective tinnitus. The selected sample consisted of 31 patients randomly assigned to the following groups: Nd:YAG laser (*N* = 15) and diode laser (*N* = 16). The laser wavelength and settings were adjusted as described by Demirkol et al. and more details were described in Table 2. The patients were exposed to the laser while seated in a dental chair with their necks supported, five times per week, for a total of 10 sessions. To assess the efficacy of LLLT, the VAS questionnaire was completed by all patients. Each patient re‐completed the VAS survey immediately after and 1 month after the end of treatment (minimum tinnitus score = 0, maximum = 10). The threshold for significance was established at 5% (*p* < .05). Above all in the Nd:YAG laser group, there were statistically significant variations between the values at the start of therapy and 1 month later (*p* = .00, and *p* = .005, respectively).

### 3.4 Prediction of outcomes indicators

Two RCTs^29^, ^33^ specifically examined prediction indicators that could indicate the success of the abovementioned orofacial therapies.

The objective of Plaza‐Manzano's study^33^ was to find out the impact of clinical, physical and psychological variables on treatment outcomes following cervico‐mandibular manual therapy associated with exercise and education. In this group of patients, the following predictor variables were found to be significantly correlated with clinical outcomes, with a *p* < .05, both at 3 and 6 months: gender, tinnitus severity, tinnitus‐related handicap, health‐related quality of life, PPTs over temporalis muscle. For instance, higher scores of tinnitus severity and tinnitus‐related handicap predicted better treatment results. In the group of patients performing exercise and education, without manual therapy, significant correlations with better clinical outcomes were only reported when tinnitus severity and PPTs showed high scores.

The article of Van der Wal, Van de Heyning et al.^29^ identified several indicators of outcomes after a 9‐week follow‐up of multidisciplinary orofacial treatment using multivariate logistic regression analysis through two prognostic models. Relevant changes in the Tinnitus Questionnaire (TQ) included as predictors the ‘duration of tinnitus’ and ‘a higher initial score on the TQ somatic subscale’. In 68.5% of patients, this model accurately predicts the result on TQ.

For the evaluation of the Tinnitus functional index score, statistically substantial improvements in TFI were predicted by ‘age,’ ‘female gender,’ and ‘duration of tinnitus’, with an accuracy of the model in 68.1% of patients.

### 3.5 Meta‐analysis

**FIGURE 3:**
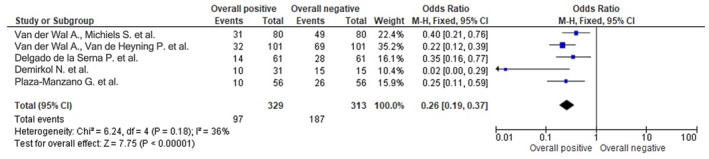
Odds ratio of selected RCTs assessing the different treatment modalities and their overall positive versus negative impact as shown on the forest plot.

**FIGURE 4:**
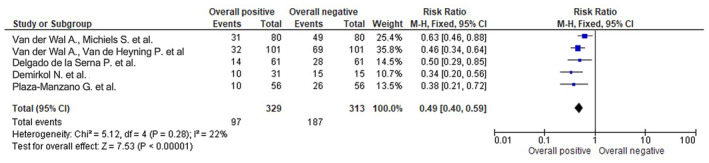
Risk ratio of selected RCTs assessing the different treatment modalities and their overall positive versus negative Impact as shown on the forest plot.

**FIGURE 5:**
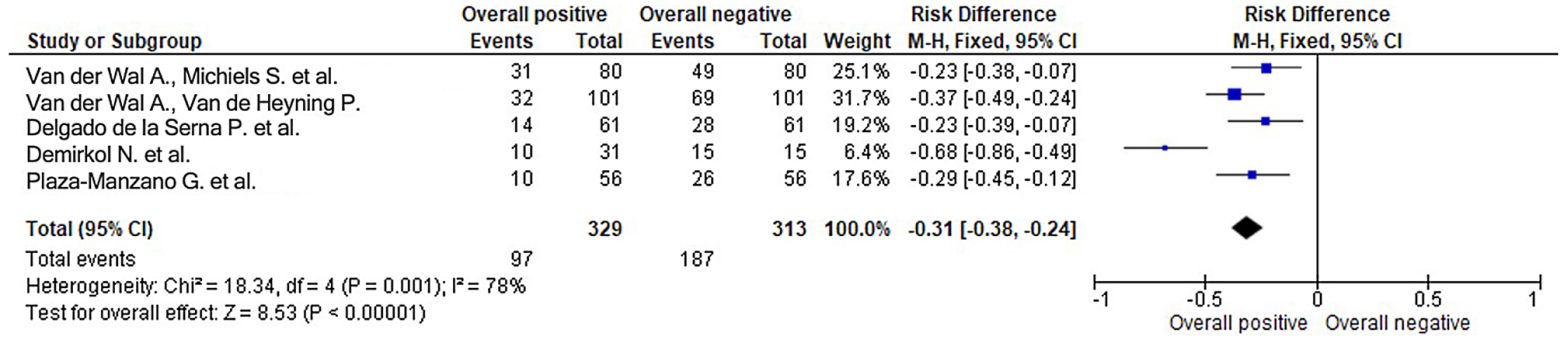
Risk difference of selected RCTs assessing the different treatment modalities and their overall positive versus negative Impact as shown on the forest plot.

Figures 3, 4, 5 respectively represent the odds ratio, risk ratio and risk difference with the utilisation of the respective treatment modalities used in the RCTs.

The forest plot in Figure 3 was used to display the results of a meta‐analysis of selected RCTs assessing different treatment modalities for tinnitus. The forest plot showed an odds ratio (OR) of 0.26 with a 95% CI of [0.19, 0.37], indicating a significant positive impact of the treatments on tinnitus symptoms. The forest plot also displayed heterogeneity statistics, with a Chi‐squared (*χ* ^2^) value of 6.24 and degrees of freedom (df) of 4, resulting in a *p*‐value of .18. The *I* ^2^ statistic, which measures the percentage of total variation across studies that is due to heterogeneity rather than chance, was calculated to be 36%. These statistics suggested moderate heterogeneity among the selected RCTs. The test for overall effect using a *Z* statistic was calculated to be 7.75 with a *p*‐value of less than .00001, indicating a highly significant overall positive effect of the different treatment modalities on tinnitus symptoms. The forest plot displayed the OR of 0.26, which represents the effect size of the treatment modalities on tinnitus symptoms. The 95% CI of [0.19, 0.37] indicated the range of uncertainty around the point estimate of the OR. The lower bound of the CI at 0.19 suggested a potential positive impact of the treatments, while the upper bound of the CI at 0.37 indicated the maximum effect size observed in the studies.

The statistical analysis for the forest plot showing RR (relative risk) of 0.49 with a 95% confidence interval (CI) of [0.40, 0.59] for selected RCTs assessing different treatment modalities and their overall positive versus negative impact is shown in Figure 4. Heterogeneity among the included RCTs was assessed using the *χ* ^2^ test, which yielded a value of 5.12 with 4 degrees of freedom (df), and a resulting *p*‐value of .28. The *I* ^2^ statistic, which quantifies the percentage of total variation across studies that is due to heterogeneity rather than chance, was calculated to be 22%. These results suggest that there is low heterogeneity among the included studies, as both the *χ* ^2^ test and *I* ^2^ statistic do not indicate significant heterogeneity (*p* > .05). The test for overall effect was performed using a *Z*‐test, which yielded a *Z*‐value of 7.53 with a corresponding *p*‐value of less than .00001, indicating a statistically significant overall effect. The forest plot displayed the RR estimates and 95% CI for each individual study, with the pooled RR estimate being 0.49 (95% CI [0.40, 0.59]), as shown on the forest plot. The forest plot visually presented the results of the selected RCTs, with the RR estimate of 0.49 indicating a significant reduction in the risk of negative impact with the use of different treatment modalities. The 95% CI of [0.40, 0.59] suggests that the true effect size is likely to fall within this range with 95% confidence.

The statistical analysis for the forest plot, which displays the risk difference (RD) for selected RCTs assessing different treatment modalities and their overall positive versus negative impact, is represented in Figure 5. Heterogeneity among the studies was assessed using the Chi‐square statistic, which yielded a value of 18.34 with 4 degrees of freedom (df), and a corresponding *p*‐value of .001. The *I* ^2^ statistic, which quantifies the proportion of total variation across studies due to heterogeneity, was found to be 78%. These results suggest the presence of substantial heterogeneity among the included studies. The forest plot displays the risk difference (RD) as the effect measure, with an estimated RD of −0.31 and a 95% CI ranging from −0.38 to −0.24. This indicates that, on average, there was a negative difference of 0.31 in the risk of the outcome of interest (e.g. positive vs. negative impact) between the treatment modalities studied. The CI provides a range of values within which the true population RD is likely to fall with 95% confidence. The test for overall effect, using the *Z* statistic, yielded a value of 8.53 with a corresponding *p*‐value of less than .00001, indicating a statistically significant overall effect. This suggests that the difference in risk between the treatment modalities assessed is unlikely to have occurred by chance alone. Overall, based on the forest plot showing the RD of −0.31 [−0.38, −0.24] for selected RCTs, there appears to be a statistically significant negative difference in the risk of the outcome of interest between the different treatment modalities studied. However, given the substantial heterogeneity observed among the included studies, a cautious interpretation of the findings is warranted.

Further research and analysis may be warranted to better understand the efficacy of different treatment modalities for tinnitus. Overall, the results of the meta‐analysis provided evidence for the positive impact of the treatments on tinnitus symptoms, supporting the potential effectiveness of these interventions in managing tinnitus in the studied population. The findings of this meta‐analysis may have important implications for clinical practice and future research in the field of tinnitus management. However, it is important to interpret these findings in the context of the limitations of the included studies and the specific population studied, and further research is needed to confirm and expand upon these findings. Overall, the forest plot and statistical analysis provided valuable insights into the impact of different treatment modalities on tinnitus symptoms in the selected RCTs.

## 4 DISCUSSION

This systematic review summarised the results reported in RCTs aimed at evaluating the different treatment approaches in patients with tinnitus associated with TMD and the possible predictive factors influencing treatment outcomes. TMD and cervical spine problems are the two most typical musculoskeletal conditions linked to somatic tinnitus.^35^ The DC/TMD^15^ have been created to facilitate TMD diagnosis and to provide a reliable and worldwide accepted template to be followed for both clinical and research purposes.^35^

For somatic tinnitus and TMD‐related symptoms, physical therapy is usually considered the best treatment. The first author to discuss this approach was Delgado De La Serna who included exercise, patient education, and manual treatment, focusing on the TMJ, cervical, and masticatory muscles in a multimodal therapy. Specifically, cervico‐mandibular manual therapy provided better results clinically (improving tinnitus‐related disability and TMD‐related disability), psychologically (improving depressive symptoms), and physically (with improvements in mandibular active range of motion).^32^ A localised hypoalgesia was reported in both groups of patients, but above all in the group treated with manual therapy, as evidenced by an increase in the pressure pain threshold (PPTs) of the masticatory muscles. These findings confirm the neuro‐physiological benefits of manual treatment and exercise on the central nervous system. The group that received manual therapy probably gained more benefits from manual contact and from time spent with the physician. All outcomes were assessed at baseline, 1 week after the treatment program, and 3/6 months after the last treatment session by an assessor blinded to group allocation.

Similarly, Van Der Wal, Michiels.^28^ documented all parameters at baseline, after 9 weeks in the delayed start group, immediately after the last treatment session, and after 9 weeks of follow‐up.

Cervico‐mandibular manual therapy was also assessed in Plaza‐Manzano's RCT^33^ and it was concluded that tinnitus severity and PPTs over the temporalis muscle at baseline were the variables most associated with poorer clinical outcomes at 3 and 6 months of treatment follow‐up. Unfortunately, some psychological variables such as anxiety, sleep quality, or patients' hopes were excluded; therefore, it was unclear how these predictor variables might influence therapy outcomes. Limitations of this RCT included the small sample size chosen and the short follow‐up.

Van Der Wal, Michiels.^28^ found positive effects on the severity of tinnitus after assessing the effectiveness of non‐invasive orofacial treatment through a delayed treatment design. The treatment protocol was designed on a maximum of 18 sessions of orofacial physiotherapy within a 9‐week program. On the other hand, Delgado de la Serna's study^32^ showed that patients underwent six treatment sessions, specifically, the first 2 during the first week, and the other once a week for a total of 1‐month treatment. LLLT in Demirkol's study^30^ was applied once per day for 10 days.

In the article of Van der Wal, Van de Heyning,^29^ ‘Female gender’ [odds ratio (OR) 2.70] and ‘a higher score on the somatic subscale of the TQ’ (OR 1.52) were the two key medical history predictors. ‘Painful palpation of the TMJ’ (OR 2.46) was obtained as a predictive indicator from the temporomandibular examination. According to audiological evaluation, a ‘better score on the speech in noise test’ (OR 0.88) was determined to be a significant prognostic predictor.

Demirkol's RCT^30^ showed that the Nd:YAG laser was more effective than the 810 nm diode laser. However, due to its higher cost, only a few studies have been conducted on the Nd:YAG laser.

In conclusion, despite the promising result of conservative TMD treatment, a myorelaxant splint associated with other therapies seems to be more efficient in tinnitus remission than treatment with a splint alone, reinforcing the effect of muscle contractures on the common structures of the auditory and stomatognathic systems.^36^

The body mass index (BMI) has not been investigated as a possible predictive factor because it was demonstrated its only marginal correlation with tinnitus.^37^ Moreover, a recent systematic review showed that obesity is not a risk factor for TMD being patients with larger BMI suffering less often from TMD pain.^38^ Other studies listed as possible causes of tinnitus, as of other TMD, the prolonged use of protective masks of different types,^20^ and the post‐COVID‐19 syndrome,^39^ but the scientific evidence is not clear.

This systematic review with meta‐analysis showed some limitations. The low number of included studies can be a limitation in the meta‐analysis. Then, there are concerns regarding the risk of bias in more than half of the evidence and there is high heterogeneity in treatment modalities. Nevertheless, the present study is the first one to investigate the treatment effects of patients diagnosed with tinnitus and related TMD according to the recent DC/TMD.^40^

It would be intriguing to assess the impact of the different treatments described in a larger sample size in multicentric prospective randomised clinical trials with longer follow‐up periods. Thus, further research in this area is warranted to better understand the optimal management strategies for tinnitus and improve patient outcomes. Future investigations in this area may also explore further factors, and potential mediators of treatment effects, comorbidities, as well as long‐term outcomes and cost‐effectiveness of different interventions.^41^ The findings of this study may have important implications for clinical practice, and in the improvement of overall patient quality of life.

## 5 CONCLUSIONS

Overall, these findings suggest that a multidisciplinary non‐invasive approach is the most efficacious treatment in adult patients diagnosed with tinnitus associated with TMDs. Baseline tinnitus severity, gender, quality of life, age and tinnitus duration were considered predictive factors of clinical outcomes in patients diagnosed with TMDs and referring tinnitus. Further research in this area will help to refine more evidence‐based treatment recommendations and optimise patient care for tinnitus management.

## CONFLICT OF INTEREST STATEMENT

The authors have no conflict of interests related to this publication.

## Data Availability

The data that support the findings of this study are available from the corresponding author upon reasonable request.
